# Type-specific seroprevalence of bluetongue in India during 2018 and 2019

**DOI:** 10.14202/vetworld.2020.2092-2096

**Published:** 2020-10-05

**Authors:** G. Naresh, Kalyani Putty, Y. Narasimha Reddy, Y. Krishna Jyothi

**Affiliations:** 1 Department of Veterinary Biotechnology, College of Veterinary Science, P. V. N. R. Telangana Veterinary University, Hyderabad, Telangana, India; 2 Department of Animal Husbandry, Andhra Pradesh Veterinary Biological Research Institute, Vijayawada, Andhra Pradesh, India

**Keywords:** Bluetongue, competitive enzyme-linked immunosorbent assay, serum neutralization test, type-specific seroprevalence

## Abstract

**Background and Aim:**

Bluetongue (BT) is a major disease of sheep and goats and is endemic to India. It is known to cause significant economic losses to the sheep industry. The current study aimed to determine the type-specific seroprevalence of BT in sheep population of India during 2018-2019.

**Materials and Methods:**

Blood samples (n=405) were collected from 6 months to 1 year old sheep from six districts (Nalgonda, Karimnagar, Khammam, Mahabubnagar, Warangal, and Ranga Reddy) of Telangana state, India. Group- and type-specific seroprevalence (against BT virus [BTV] serotypes BTV-1, 2, 4, 5, 9, 10, 12, 16, 21, 23, and 24) was studied by competitive enzyme-linked immunosorbent assay and serum neutralization test, respectively.

**Results:**

Results showed an overall seroprevalence of 14.81% (n=60) with the highest seroprevalence of 50% in Khammam district. Seroprevalence of BTV-1, 2, 4, 5, 9, 10, 12, 16, 21, 23, and 24 was noted as 16.66%, 11.66%, 31.66%, 11.66%, 05%, 6.66%, 16.66%, 8.33%, 13.33%, 6.66%, and 16.66%, respectively. The majority of the sera neutralized more than 1 serotype, indicating superinfection or circulation of multiple serotypes in the sampled flocks. This mixed seroprevalence was observed in 43.33% of the sera with number of BTV serotype-specific antibodies ranging from two to eight in individual animals.

**Conclusion:**

Regular monitoring of circulating serotypes, especially in young herds, elucidates pattern of dominating serotypes in a particular area during a season. This knowledge can be applied to design appropriate vaccination strategies by including particular serotypes of virus as part of a multivalent vaccine for a particular period, in a particular area.

## Introduction

Bluetongue (BT) is a major Office International Des Epizooties listed disease of sheep and goats and is endemic to India, causing significant economic losses to the sheep industry [1]. It is an insect-borne viral disease of ruminants characterized by inflammation of mucous membranes, congestion, swelling, and hemorrhages. The causative agent is BT virus (BTV) of the genus *Orbivirus* in the family Reoviridae. The virus is transmitted between vertebrate hosts by *Culicoides* species [2,3]. Outbreaks are frequently reported in the southern states of India, especially during monsoon (July-September) when the activity of the vector is highest and is hence propitious for the occurrence of BT. At present, a total of 29 serotypes of BTV were recognized worldwide [4]. In India, at least 24 serotypes have been identified based on serology and/or virus isolation [5-8]. In India, a pentavalent inactivated vaccine containing BTV serotypes BTV-1, 2, 10, 16, and 23 are commercially available (M/S Indian Immunologicals, Hyderabad) for field usage [9].

Infection with multiple serotypes/topotypes of the virus is common in enzootic areas, which makes the control of BT difficult [10]. To design improved prevention and control strategies, it is important to determine the major circulating serotypes of the virus.

With this background, as a first step to reveal type-specific seroprevalence of BT in India during 2018-2019, the present study was undertaken to assess type-specific anti-BTV antibodies in sheep of different parts of Telangana state which rank top in the country in sheep population and also where the disease is rampant with major economic losses experienced. Sheep of age 6 months-1 year were selected for sampling as they represented a group that might have been exposed to only one season of monsoon and, therefore, one season of BT.

## Materials and Methods

### Ethical approval

As per the Committee for the Purpose of Control and Supervision of Experiments on Animals guidelines, a study involving collection of clinical samples under field conditions does not require approval of Institute Animal Ethics Committee. Blood samples were collected by licensed veterinarians as per standard sample collection methods without any harm or stress to the animals.

### Study area

Telangana is one of the southern states of India situated between 16 and 20° latitude and between 77.5 and 81.5° longitudes and is situated on the Deccan plateau in the central stretch of the eastern seaboard of the Indian Peninsula. It is a semi-arid area and has a predominantly hot and dry climate. Summers start in March and peak in May with average high temperatures in the 42°C (108°F)-45°C (113°F) range. The monsoon arrives in June and lasts until September with about 755 mm (29.7 inches) of precipitation. A dry, mild winter starts in late November and lasts until early February with little humidity and average temperatures in the 22-23°C (72-73°F) range. The annual rainfall is between 900 mm and 1500 mm in North Telangana and 700 and 900 mm in South Telangana, from the southwest monsoons.

### Collection of sera samples

**Figure-1:**
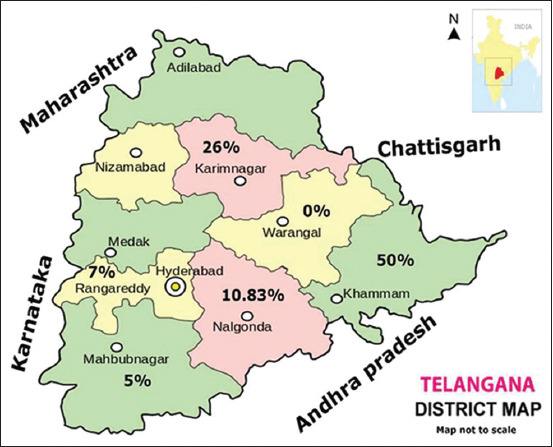
Overall seroprevalence of bluetongue in different districts of Telangana. Seroprevalence was studied in six districts of Telangana state: Karimnagar, Warangal, Khammam, Nalgonda, Mahabubnagar, and Ranga Reddy. Overall seroprevalence in each district was indicated as percentages in the map [Source: https://www.uokpl.rs/rsvi/hTboixb_ancient-telangana-tourist/].

The samples were collected during December 2018-March 2019. A total of 405 blood samples were collected from sheep between the ages of 6 months and 1 year from selected villages of six districts (Nalgonda, Karimnagar, Khammam, Mahabubnagar, Warangal, and Ranga Reddy) in Telangana state (Figure-1). The animals belonged to nomadic marginal farmers, were not maintained in organized farms (with no apparent sire and dam data/history), and were not vaccinated with the commercial inactivated pentavalent vaccine of BT. The blood samples were collected in Vacutainer tubes without ethylenediaminetetraacetic acid or heparin. From blood samples, sera were separated and stored at −20°C till further use at the Department of Veterinary Biotechnology, College of Veterinary Science, Hyderabad, where all the laboratory tests were performed.

### Group-specific seroprevalence of BT

The test sera were subjected to competitive enzyme-linked immunosorbent assay (c-ELISA) to detect antibodies against the most conserved viral protein, VP7 of BTV, using a commercial kit (Veterinary Diagnostic Technology, Inc., USA). As per the manufacturer’s instructions, percentage inhibition values >50 were considered as positive. Those that fall between 40% and 55% were suggested to be retested. Two samples of the total sampled in the current study were in the retest range (45%). Those on retesting remained in the negative result group.

### Type-specific seroprevalence of BT

Plaque purified viruses BTV-1, 2, 4, 5, 9, 10, 12, 16, 21, 23, and 24 were used in the serum neutralization assay for detecting serotype-specific antibodies. These viruses were the only BTV isolates available with us and were isolated from previous BT outbreaks in the state (unpublished data). Hyperimmune sera against each of the plaque purified BTV serotypes were used as positive controls with no apparent cross-reactivity between serotypes. Hyperimmune sera for the tested BTV serotypes were raised by us previously as follows: BTV seronegative Deccani sheep (6-8 months) were shifted to insect proof experimental animal house. After deworming and acclimatization, 1 ml of 10^6^/ml TCID50 virus was inoculated to sheep by two routes, that is, 0.5 ml through intradermal and 0.5 ml through subcutaneous routes at shoulder region. On the 15^th^ day of infection, one more injection of same dose was given to the animals. Serum samples were collected on 0, 7, 14, 21, and 28 days post-inoculation to assess the antibodies level and seroconversion. For type-specific seroprevalence, the SNT method was as follows: 50 μl consisting of 100 TCID_50_ of individual BTV serotype was incubated with 50 μl of 1:20 diluted serum for 1 h at 37°C. The serum-virus mixture was then inoculated on to confluent Vero cell monolayers in tissue culture plates, in quadruplicate. Cells were observed for cytopathic changes for 5 days at 24 h interval. After 5 days, the culture plates were fixed with 10% formaldehyde and stained with 1% crystal violet (HiMedia, Mumbai). Wells with 25-100% staining were considered to have no or limited continuing professional education (CPE) (i.e., positive for neutralization). Wells with <25% staining were considered to have extensive or full CPE (i.e., negative for neutralization).

## Results

### Group-specific seroprevalence of BT

The sera were first screened by c-ELISA for the detection of BTV group-specific antibodies. Out of 405 serum samples, 60 were found positive for BTV group-specific antibodies giving a positive percentage of 14.81%. The seropositivity of BTV in the sheep was 10.83%, 26.0%, 50.0%, 5.0%, 0%, and 7.0% for Nalgonda, Karimnagar, Khammam, Mahabubnagar, Warangal, and Ranga Reddy districts of Telangana state, respectively (Figure-1).

### Type-specific seroprevalence of BT

c-ELISA-positive samples were screened for type-specific neutralizing antibodies against BTV serotypes BTV-1, 2, 4, 5, 9, 10, 12, 16, 21, 23, and 24. Out of the 60 positive sera, 19 (31.66%) showed antibodies against BTV-4, which was highest for the study period, followed by 10 (16.66%) to BTV-1, -12, and -24 each. Further, 8 (13.33%) were positive for BTV-21; 7 (11.66%) for BTV-2 and -5 each; 5 (8.33%) for BTV-16; 4 (6.66%) for BTV-10 and -23 each, and 3 (5%) to BTV-9 (Table-1). Mixed seroprevalence with each individual animal harboring BTV antibodies raised against more than one viral serotype was observed in 43.33% of BTV group-specific seropositive animals. The number of serotypes varied from two to eight with a variety of serotype combinations (Table-2).

**Table 1:** Type-specific seroprevalence of BTV.

S. no.	Districts	c-ELISA positive %	Prevalent BTV serotypes
1.	Nalgonda	10.83 (13/120)	1, 2, 4, 5, 10, 12, 16, 21, 23, and 24
2.	Karimnagar	26 (13/50)	1, 2, 4, 5, 12, 16, 23, and 24
3.	Khammam	50 (25/50)	1, 2, 4, 5, 9, 10, 12, 21, and 24
4.	Mahabubnagar	05(2/40)	1, 2, 4, 5, 9, 12, 16, 21, and 24
5.	Warangal	0 (0/45)	-----------
6.	Ranga Reddy	07 (7/100)	4, 5, 12, 21, and 24
	Total	14.81% (60/405)	11 serotypes

BTV=Bluetongue virus, c-ELISA=Competitive enzyme-linked immunosorbent assay

**Table 2:** Number and types of mixed serotype seroprevalences observed in Telangana state, India, during 2018-2019.

S. no.	Number serotypes	BTV serotype combinations
1.	Two serotypes	BTV-1 and 2, 1 and 4, 1 and 9, 2 and 21, 4 and 5, 4 and 12, 4 and 24, 9 and 12, 9 and 24, 10 and 23, 12 and 21
2.	Three serotypes	BTV-5, 21, and 24; 10, 16, and 23; 12, 16, and 23; 1, 4, and 24; 2, 4, and 24
3.	Four serotypes	BTV-1, 2, 4, and 24; 1, 2, 4, and 5; 4, 10, 12, and 21
4.	Five serotypes	BTV-10, 12, 16, 21, and 23
5.	Six serotypes	BTV-1, 2, 4, 5, 21, and 23
6.	Eight serotypes	BTV-1, 2, 4, 5, 12, 16, 21, and 24

BTV=Bluetongue virus

## Discussion

BTV seroprevalence of varying percentages was reported by several authors before [11-17]. In BT endemic countries such as India, high seroprevalence (>50%) is usually reported [18-22]. Especially, in the tested region included in the present study, the previous studies reported 61% seroprevalence of BTV during 2005-2009 and 75% during 2014-2017 [5,23,24]. Here, we report a comparatively low seroprevalence of BTV than was reported before. Such low rates (9.3-18.4%) were also reported by other researchers [25-27]. A huge variation between the findings of various authors regarding seroprevalence of BTV among small ruminants in various states of India may be due to varied climatic conditions, sheep population density, and susceptibility of sheep breeds to BT. In the current study, age (6 months-1 year old) of the sheep selected might have played a major role in the results observed. Young animals have a lesser probability of exposure to BTV infection as they would have been exposed to fewer monsoon seasons where peak *Culicoides* activity is noticed. In spite of this reason, young animals were chosen in the study to identify the circulating serotypes in the current season. This could also be a major reason in 0% BTV seroprevalence found in one of the districts included in the current study. Attempts at isolating BTV from the samples collected were not successful (data not shown), probably because blood was collected from apparently healthy animals which might have had exposure to BTV infection much earlier and viremia cleared before the samples were collected. In the current study, only one dilution (1:20) of the test serum was used in the neutralization assay. Titration of antibodies was not done in the present study as our main aim was to identify the circulating serotypes of BTV. However, serotype titer ranges (low, medium, or high) per area would have provided a better understanding of the infection severity and seroprevalence in the tested region.

India being endemic to BT, it is not uncommon to find such mixed serotype seroprevalence [5]. This indicates superinfection or circulation of multiple serotypes in the sampled flocks, which reflected in the multiserotype virus isolation from individual BT suspected animals [6]. In Telangana state specifically, a vast variation in the serotype prevalence over the past decade in different livestock hosts was previously observed [5,23,24,28] (summarized in Table-3). On comparison, it can be noted that, during 2005-2009, seroprevalence of BTV-1 and 2 was high; seroprevalence of BTV-16, 21, and 9 was dominant during 2014 and 2015; BTV-23, 1, and 16 during 2015-16; BTV-2, 1, and 4 during 2016-2017; and during 2018-2019, seroprevalence of BTV-4, 1, 12, and 24 was high (summarized in Table-3). Meanwhile, in the Northeastern part of India, during 2014-2017, type-specific seroprevalence of BTV-1, 16, and 10 was reported in high rates in goats. It is interesting to note from these findings, emergence of serotypes not included in the commercial pentavalent inactivated BT vaccine (harboring BTV-1, 2, 10, 16, and 23), that is, BTV-4, 9, 12, 21, and 24.

**Table 3:** Type-specific seroprevalence of BTV in various regions during different periods in India.

S. No.	Region	Species	Prevalence
1.	Andhra Pradesh and Telangana (2005-2009) [5]	Sheep	50.0% of BTV-1
			44.23% of BTV-2
			26.92% of BTV-10
			21.25% of BTV-9
			15.38% of BTV-23
			0% of BTV-21
2.	Andhra Pradesh and Telangana (2014-2015) [23]	Sheep, goat, and calf	70% of BTV-16
			30% of BTV-21
			25% of BTV-9
			12.5% of BTV-2
			7.5% of BTV-10
			2.5% of BTV-23
3.	Andhra Pradesh and Telangana (2015-2016) [23]	Sheep, goat, buffalo, and cattle	25.5% of BTV-23
			18% of BTV-1
			17.4% of BTV-16
			11.1% of BTV-2
			6.2% of BTV-21
			4.3% of BTV-12
			2.5% of BTV-4
			1.2% of BTV-9
4.	Andhra Pradesh and Telangana (2016-2017) [24]	Sheep and goat	40.62% of BTV-2
			34.37% of BTV-1
			28.12% of BTV-4 and 16
			25% of BTV-24
			9.37% of BTV-23
			6.25% of BTV-9 and 12
			3.12% of BTV-21
5.	Tripura (2014-2017) [28]	Goats	65.27% of BTV-1
			26.38% of BTV-16
			20.83% of BTV-10
			13.88% of BTV-9 and 23
			6.94% of BTV-2
6.	Telangana (2018-2019) [Current study]	Sheep	31.66% of BTV-4
			16.66% of BTV-1, 12 and 24
			13.33% of BTV-21
			11.66% of BTV-2 and 5
			8.33% of BTV-16
			6.66% of BTV-10 and 23
			5.0% of BTV-9

BTV=Bluetongue virus

## Conclusion

The current study indicates that BTV circulation is widespread in Telangana state of India. We have also observed seroprevalence of different BTV serotypes along with the presence of multiple seroprevalences in individual animals. In comparison with the previous reports, it may be concluded that different BTV serotypes were predominant during different periods in a particular region, along with the presence of mixed serotype prevalences. Surveillance programs based on seromonitoring can help forecast possible future outbreaks. For establishing effective control strategies like vaccination, extensive surveillance in different regions of the country is crucial.

## Authors’ Contributions

KP and YNR supervised, designed, and coordinated the study. GN and YKJ collected samples and performed the experiments. KP, and YNR analyzed the data. KP and YKJ wrote the manuscript. All authors read and approved the final manuscript.
